# Substance use risk and controlled substances discussions in cancer care: Observations from audio-recorded patient–clinician encounters

**DOI:** 10.1017/S1478951526102739

**Published:** 2026-06-15

**Authors:** Miryam Yusufov, Kathryn I. Pollak, Hanneke Poort, Yvan Beaussant, Elise C. Tarbi, Richard E. Leiter, James A. Tulsky

**Affiliations:** 1 Department of Supportive Oncology, Dana-Farber Cancer Institute, Boston, MA, USA; 2 Harvard Medical School, Boston, MA, USA; 3 Cancer Prevention and Control, Duke Cancer Institute, Durham, North Carolina, USA; 4 Department of Population Health Sciences, Duke University School of Medicine, Durham, North Carolina, USA; 5 Department of Nursing, University of Vermont, Burlington, Vermont, USA

**Keywords:** Behavioral medicine, cancer care, controlled substances, pain, stigma, substance use

## Abstract

**Objectives:**

Patients with advanced cancer frequently experience pain and psychological distress, often requiring controlled substances such as opioids and benzodiazepines. Although access to these medications increases risk of substance misuse, little is known about how clinicians and patients discuss controlled substance use during cancer care. Understanding these conversations may inform safer prescribing, improve patient outcomes, and support management of substance use disorder (SUD) risk. We aimed to characterize discussions of controlled substance use in oncology visits, including who initiated conversations, clinician responses, and verbalized SUD risk factors.

**Methods:**

Five coders reviewed 826 audio-recorded oncology visits from a prior clinical trial. Encounters were coded for substance type, initiator (patient, clinician, both, neither), clinician/patient response style (avoidant, concerned/emotional, engaged, neutral, resistant), and substance misuse risk factors.

**Results:**

Mean patient age was 59.5 years; most were female (55.8%), White (81.7%), and married (71.7%). Substance-related content appeared in 14.6% of counters (*n* = 121; 92 unique patients). Mentioned substances included opioids and sedative-hypnotics (benzodiazepines/sleep aids), with oxycodone referenced in 67 visits. Patients initiated discussions more frequently (*n* = 51) than clinicians (*n* = 33), though not significantly, *χ*^2^(1, *N* = 95) = 33.00, *p* = 0.078. For the remaining encounters, neither initiated (n= 24) or both initiated (n=13). Among patient-initiated discussions, clinician response types were engaged (*n* = 25), neutral (*n* = 10), avoidant (*n* = 12), concerned/emotional (*n* = 1), or resistant (*n* = 3). Common substance misuse risk factors included inadequate pain management (*n* = 28), medication concerns (*n* = 17), dose escalation (*n* = 11), psychological concerns (*n* = 11), and substance misuse/drug-seeking (*n* = 5).

**Significance of results:**

Despite widespread prescribing of controlled substances in oncology, discussions remain infrequent, and clinician responses to SUD-related concerns are often insufficient. These findings highlight opportunities to improve communication and risk management in cancer care.

## Introduction

Patients with advanced cancer often suffer from pain and psychological distress (Zabora et al. 2001; Lee and Singh 2021; Bates et al. 2022; Evenepoel et al. 2022). To manage these challenges, clinicians frequently prescribe controlled substances, including opioids (Greer et al. 2012) and sedative hypnotics (benzodiazepines/sleep aids). Opioids play a central role in cancer pain management with 60–90% of patients with advanced cancer being prescribed opioids (Greer et al. 2012) as 70–90% of such patients experience substantial pain requiring opioid therapy (Institute of Medicine, 2011). Recent data from Veterans Affairs (VA) and non-VA settings reinforce these findings. Collectively, these studies underscore the widespread reliance on opioids in cancer care.

In addition to pain management, patients with advanced cancer often struggle with anxiety and sleep impairment; clinicians may respond by prescribing controlled substances (e.g., benzodiazepines, sleep aids) – sometimes even beyond active treatment. For example, studies suggest a higher prevalence of benzodiazepine prescriptions in cancer samples, compared to non-cancer samples (Haque et al. 2021; Sakamoto et al. 2021). Further, a population-based study using SEER Medicare Data found that 13% of older women with breast cancer had concurrent prescriptions for opioids and benzodiazepines within a year post-diagnosis (Sakamoto et al. 2021). Finally, a retrospective chart review of 124 women undergoing chemotherapy found that 32.3% were prescribed sleep aids, with lorazepam (31.4%) and zolpidem (29.4%) being the most commonly prescribed (Haque et al. 2021). Pain and side effects of cancer treatment impair the lives of patients with cancer. Treating these symptoms with controlled substances carries risks. Exposure to benzodiazepines and opioids increases risk of adverse events, such as overdose, falls or fractures, or all-cause hospitalization, in older adults with cancer (Roberts et al. 2021).

Although oncology and palliative care clinicians routinely prescribe and manage controlled substances during cancer care (and often encounter patients with substance use concerns) (Childers et al. 2015), they frequently receive insufficient training and institutional support (e.g., referral pathways, guidelines, psychosocial oncology/addiction expert consults, etc.) to effectively assess substance use risk and establish appropriate clinical boundaries (Merlin et al. 2019). Given that patient exposure to controlled substances amplifies substance use disorder (SUD) risk, it is important to address this clinically. For example, the Proove Opioid Risk study found that individuals at moderate risk had 4.17 times increased odds of being diagnosed with opioid use disorder, compared to control and those in the high-risk category had 16.5 times increased odds (Brenton et al. 2017). Similarly, a large-scale retrospective study found that long-term benzodiazepine use in patients with anxiety disorders was associated with a 3.00 times higher risk of SUDs compared to non-users (Sun et al. 2024). Of note, neither of these studies were conducted with patients with cancer, despite prior research suggesting inadequate support for prescribing clinicians. A study of 6,101 cancer survivors found that SUD was most prevalent in survivors of head and neck, esophageal/gastric cancer, cervical cancer, and melanoma (Jones et al. 2024). Nonetheless, concern about SUD risk should not deter clinicians from prescribing controlled substances when clinically indicated, particularly in patients with limited prognosis, where effective pain/symptom management is critical.

Despite the prevalence of substance use concerns across the cancer trajectory (Yusufov et al. 2019), clinician training in detecting and managing SUD remains limited. Across 34 hospice and palliative medicine programs, 77.2% of fellows treated at least 1 patient with an SUD and 43.9% treated a patient misusing opioids (Childers et al. 2015). Further, a study of 167 physicians and nurse practitioners from national palliative care organizations revealed that only 27% reported having training or systems in place to address substance misuse in patients with cancer (Merlin et al. 2019). In sum, despite rising awareness of SUDs and non-medical use during cancer care (Yusufov et al. 2019, 2023, 2024), there remains a significant lack of data to guide patients and clinicians in effectively navigating appropriate use and potential misuse.

### Purpose of present study

Because SUD risk can only be identified and addressed when it is openly discussed, clinician–patient communication about controlled substances is critical. Yet, it remains unclear how often these discussions occur in oncology settings, who initiates them, and how clinicians respond when concerns arise.

To assess the prevalence and nature of patient–clinician communication regarding substance use, we analyzed audio-recorded patient-oncology clinician outpatient visits. We identified the prevalence of substance use-related content, whether it was clinician or patient-initiated, and how substance use-related concerns were handled within the encounters. Specifically, we were interested in how oncologists responded to substance use concerns in patients with advanced cancer. Informed by the current literature (Childers and Arnold 2012; Yusufov et al. 2023, 2024), our central hypothesis was that fewer than half of the encounters would contain content related to substances – and that fewer than half of those encounters would contain adequate management of substance use concerns (e.g., early refill requests, addiction worries, etc.), such as referrals, empathic statements, and boundary-setting. Our overarching goal was to identify and describe: (1) how content related to controlled substances arises; and (2) how oncology clinicians manage this content in encounters with their patients.

## Methods

### Data source and sample

This analysis used audio-recorded clinical encounter data from the Communication in Oncologist-Patient Encounters (“COPE”) trial, an intervention study designed to help patients express emotional concerns to their oncology clinicians. About 343 patients enrolled in oncology clinics at Duke Medical Center (*n* = 232) and the University of Pittsburgh Medical Center (*n* = 111) were recorded in a total of 826 encounters with their cancer clinicians (oncologists or advance practice clinicians, such as nurse practitioners or physician assistants) (Porter et al. 2015). This study received respective Institutional Review Board (IRB) approvals (Duke IRB #PRO 00013032; University of Pittsburgh #PRO 09020116) and study patients provided *written consent* to audio recordings during their visits.

### Coding procedure

During the coding process for the primary trial analysis (which focused on empathic opportunities and clinician responses), each coder identified the prevalence of content related to substance use for the purpose of this additional study. The operational definition of “substance use content” was “mention of controlled substances, such as opioids, benzodiazepines, or sleep aids, or substance use-related concerns during the clinician–patient encounter,” as well as pain that involved an affective component (e.g., patient mentions emotional struggles, functional impairment, etc.). Five independent coders (MD and PhD-level clinicians) reviewed 826 audio-recorded oncology clinician–patient encounters for substance use-related content, and 144 (17.4%) of these encounters were flagged for containing content broadly related to controlled substances. Across all code types, interrater reliability was high, ranging from *k* = 88.13 to *k* = 100. Coding for this study was conducted collaboratively using AVAnalyze (formerly available at avanalyze.com), a web-based platform that supported team-based annotation and analysis.

### Data analysis

This study is a secondary analysis of audio-recorded outpatient oncology encounters collected in 2015 as part of a clinical trial of patient-oncology clinician communication. The parent study was not specifically designed to examine controlled substance use or prescribing practices. As such, detailed clinical data regarding patients’ active prescriptions, including whether patients were currently prescribed opioids or other controlled substances at the time of the encounter, were not systematically collected. Analyses are, therefore, limited to substance-related content explicitly verbalized during the recorded encounters. To verify substance-related content from the audio recordings, we reviewed the transcripts of all 144 clinician–patient encounters that were originally flagged as having “substance use content” during the coding phase. We extracted which controlled substances were mentioned during each encounter. We then subcategorized each encounter with substance use-related content into “clinician-initiated content,” “patient-initiated content,” “both,” or “neither.” If a controlled substance was named during routine medication review but neither the clinician nor patient engaged in further discussion (e.g., use, effects, concerns), the encounter was coded as “neither” initiating the topic but still included due to the substance mention. Finally, we coded response characterization (i.e., reflecting how the other party responded to the substance-related topic. Responses were coded into the following categories: (1) engaged (i.e., active participation, asked questions, collaborative discussion); (2) avoidant (i.e., deflecting, changing the topic, minimizing); (3) neutral (i.e., no response, informational, or logistical responses without emotional tone); (4) concerned/emotional (i.e., displays of empathy, concern, or distress); and (5) resistant (i.e., disagreement, skepticism, or reluctance to proceed). We also identified risk factors for SUD across all encounters that contained substance use content (*n* = 121). Known risk factors include inadequate pain control (Voon et al. 2018), medication/addiction concerns, dose escalation (Hayes et al. 2020), psychological concerns (with accompanying coping skills deficits) (McHugh & Otto, 2012; Kneeland et al. 2019; McHugh and Kneeland 2019), and medication misuse and/or drug-seeking (Boyd et al. 2018).

### Statistical methods

**Figure 1.**
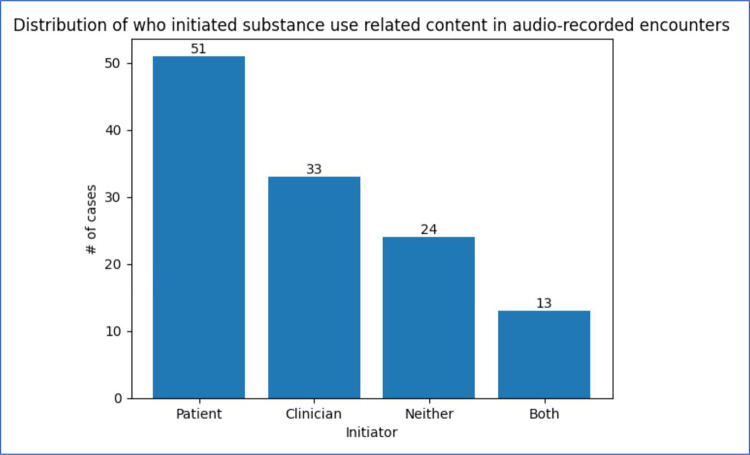
Distribution of who initiated substance use-related content in audio-recorded encounters. Figure 1 long description.

*Notes.* The above figure demonstrates that across 121 encounters, patients initiated content related to controlled substances 51 times and clinicians initiated content in 33 encounters. In 24 cases, neither the patient nor clinician-initiated content related to controlled substances, despite the encounter being flagged for mention of a controlled substance. In 13 encounters, both, the clinician and patient-initiated content related to controlled substances.

We used descriptive statistics to quantify controlled substance types, summarize who initiated substance-related discussions (i.e., clinician, patient, both, neither) (Figure 1), how the other party responded (engaged, avoidant, neutral, concerned/emotional, resistant), and identified prevalence of observed risk factors. Definitions were as follows: (1) “engaged” = actively participates, asks questions, agrees to plan, or discusses options; (2) “avoidant/deflecting” = changes the topic, minimizes the issue, or avoids responding meaningfully; (3) “neutral” = acknowledges the topic without strong emotional/behavioral reaction; (4) “concerned/emotional” = expresses fear, frustration, distress, or emotional discomfort; and (5) “resistant” = pushes back, questions necessity, expresses doubt, declines recommendations. To visualize communication dynamics, we generated a stacked bar chart showing response types stratified by initiator (clinician, patient, both). Encounters categorized as “neither” were excluded from this visualization due to absence of an active participant.

To assess whether patients or clinicians were more likely to initiate discussions, we performed McNemar’s test using paired data from discussion codes where both initiator roles could be evaluated. All instances marked as initiated by “Neither” were excluded from analysis. This left a sample of 95 codes with “patient” or “clinician” initiators. Statistical analyses were conducted using ChatGPT 2025 version using the prompt “Using the dataset above, run a McNemar’s test to determine whether clinicians or patients initiated more discussions, after deleting all ‘neither’ cases.” – and reviewed for accuracy. Descriptive statistics for patient demographics were run using the Statistical Package for Social Sciences version 28.0 (SPSS v. 28.0). Analyses were conducted at the encounter level. Although some patients (26%) contributed to multiple encounters, the majority (74%) contributed one; therefore, clustering by patient was not modeled.

## Results

The mean age of the final analytic sample (patient level data; *N* = 92) was 60.6 (SD = 11.7). Most participants were female (56.5%), White (80.4%), and married (72.8%). See Table 1.

**Table 1.** Demographic characteristics (*N* = 92 unique patients) Table 1 long description.

Patient characteristic	Mean (SD) or frequency (%)
**Age (years)**	59.5 (SD = 11.8)
**Sex**	
Female	52 (56.5)
Male	40 (43.5)
**Race/ethnicity**	
Non-Hispanic White	74 (80.4)
Black	15 (16.3)
Asian	2 (2.2)
American Indian/Alaska Native	1 (1.1)
Hispanic	0
**Marital status**	
Married	67 (72.8)
Divorced/Separated	16 (17.4)
Widowed	5 (5.4)
Never married	4 (4.3)
**Education**	
Completed high school/GED	17 (18.5)
Some college	26 (28.3)
Completed college	32 (34.8)
Graduate school	17 (18.5)

*Note*. Demographic information was missing for 1 encounter.

Among the 92 unique patients who generated a total of 121 substance use-related encounters, most (73.9%) contributed a single encounter, while 21.7% contributed 2 encounters, and 4.3% contributed 3 encounters.

Of the full sample of oncology clinician–patient encounters (*N* = 826), coders flagged 144 (17.43%) encounters as “substance use encounters,” meaning that they contained content broadly related to controlled substances. After deleting encounters that were erroneously flagged in that they either had no substance use content or were flagged because of substances that are not controlled (e.g., gabapentin) (*n* = 23), our final analytic sample was 121 encounters. Opioids, benzodiazepines, and sleep aids were the 3 classes of controlled substances represented in this dataset, with oxycodone being the most frequently mentioned (*n* = 67). See Table 2.

**Table 2.** Controlled substances discussed Table 2 long description.

Medication	Mentioned in *N* encounters
**Opioids**	
Oxycodone	67
OxyContin	30
Percocet	12
Morphine	11
Dilaudid (hydromorphone)	7
Hydrocodone	6
Fentanyl	6
Vicodin	5
Methadone	3
Tramadol	1
Tapentadol	1
Demerol	1
**Benzodiazepines**	
Ativan (lorazepam)	14
Xanax (alprazolam)	7
Klonopin (clonazepam)	4
Valium (diazepam)	2
**Z-drugs and sleep aids**	
Ambien (Zolpidem)	14

*Notes*. In 8 of the encounters, “pain meds” were discussed with no reference to which pain medication; medications not mutually exclusive, in some cases, there were co-prescriptions such as oxycodone and OxyContin.

**Figure 2.**
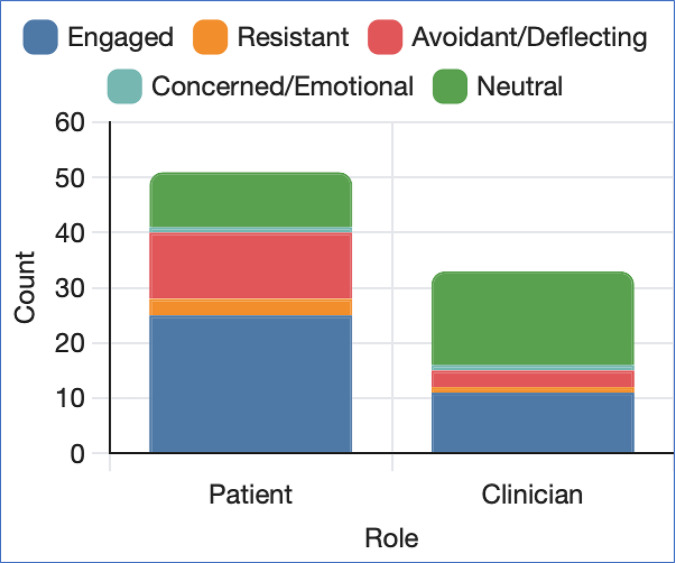
Differences in response types by initiator (patient/clinician) across encounters. Figure 2 long description.

*Notes.* This figure demonstrates frequency with which clinicians and patients initiated substance use-related encounters. Of the 121 encounters, 24 encounters had neither patient nor clinician initiate substance-related counters – these encounters were excluded from the figure. When both, patient and clinician initiated (*N* = 13), we recorded 2 separate response instances (1 for each initiation) because each initiation may have elicited its own kind of response (e.g., neutral vs. avoidant).

Among the 121 encounters in which controlled substances were discussed, patients initiated discussions in 51 cases (42.1%), clinicians initiated in 33 cases (27.3%), both initiated in 12 cases (9.9%), and neither initiated in 25 cases (20.7%) (see Figure 1). McNemar’s exact test revealed no statistically significant difference between the likelihood of patients and clinicians initiating discussions, *χ*^2^ (1, *N* = 95) = 33.00, *p* = 0.078. While patients more frequently initiated discussions independently (*n* = 51) compared to clinicians (*n* = 33), the observed difference did not reach statistical significance using the *α* = 0.05 threshold. Among patient-initiated discussions, clinician response types were engaged (*n* = 27), neutral (*n* = 19), avoidant (*n* = 5), concerned/emotional (*n* = 2), or resistant (*n* = 2) (Figure 2).

Of note, “Neither” cases were excluded from this analysis (McNemar’s exact test) and “Both” cases were handled as follows: when both, patient and clinician initiated, we recorded 2 separate response instances (1 for each initiation) because each initiation may have elicited its own kind of response (e.g., neutral vs. avoidant) (see Figure 2).

### Risk factors for SUD

In these data, the prevalence of observed risk factors was as follows: inadequate pain management (*n* = 28), medication concerns (*n* = 17), dose escalation (*n* = 11), psychological concerns (*n* = 11), and substance misuse and drug-seeking (*n* = 5). Of the 121 encounters, 67 had no observed risk factors. For an overview of risk factor examples identified across the patient–clinician encounters, see Table 3.

**Table 3.** Initiation and response pattern examples Table 3 long description.

Risk factor content category	*N* (%)	Initiator	Example quotes	Response	Response type
Inadequate pain management	33 (27.5)	Patient	“Sounds like you’ve been back to the pain clinic a couple of times.”	Patient explains that Dilaudid and oxycodone don’t help.	Engaged
		Clinician	“I can also give you some oxycodone. Your pain is making me concerned.”	“I actually tried one from [family member] … so much pain”	Emotional/concerned
Medication/addiction concerns	20 (16.7)	Patient	Don’t want any more OxyContin. My son has an addiction problem and he stole my OxyContin. Oxycodone is fine.	Asks how patient is managing without it; offered therapy referral.	Engaged
		Patient	“So much oxycodone … going to turn into a drug addict”	No response	Avoidant
		Patient	Taking more Dilaudid than usual	Clinician provides refills	Neutral
Dose escalation	15 (12.5)	Patient	“You know, with my doing extra stuff, I need extra pills. So, when last time it was prescribed for 180 pills for 3 months, I really blew through those.”	“Yeah, so unfortunately, I have to stick with that number.”	Resistant/boundary setting
Psychosocial concerns	13 (10.8)	Patient	So emotionally drained and depressed … all of the negative thoughts entered my mind.	“It does sort of convince you that you need the medicine.”	
Substance misuse	5 (4.1)	Patient	“Combining alcohol with Ativan helps me sleep”	No response	Avoidant

Below are example encounters between clinicians and patients regarding controlled substances. Of note, some of this content is presented in Table 3. The encounters below demonstrate back-and-forth communication between clinicians and patients, rather than singular responses to an initiation (Table 3).

**Encounter #1: Patient-initiated and clinician responded with concern**

Patient: *I just wanted to tell you that occasionally, I take Percocet. And then I go, “Is that just a regular headache? You know, that’s the kind of things I do. That’s how I think.”*

Clinician: *I think it’s time to begin de-escalating that.*

**Encounter #2: Patient-initiated and clinician avoided**

Patient: “*So much oxycodone … going to turn into a drug addict*.”

Clinician: *No response.*

**Encounter #3: Patient-initiated and clinician dismissed concerns**

Patient: “*I always thought these pain killers are always a dangerous thing, you know*.”

Clinician: “*Well, not when you have pain, though*.”

**Encounter #4: Patient-initiated and clinician engaged**

Patient: *… because I’ve always told you that oxy was always my concern. I don’t want –*

Clinician: *The oxycodone.*

Patient: *Yeah, I kind of –*

Clinician: *And I told you that’s something temporary. If you have symptoms, we have to manage them. I have no concern that you took oxycodone because you’re taking it only for pain. So, there’s no concern you’re going to get addicted to it or anything.*

**Encounter #5: Patient-initiated and clinician engaged**

Patient: *All I needed was that oxycodone and OxyContin.*

Clinician: *I know. They said you were going to become addicted to it. I was like “he’s got pancreatic cancer.”*

Patient: *I’m already – yeah, I think I’m addicted to something.*

Clinician: *You’re not addicted; you’re dependent upon it.”*

Patient: *As soon as my wife started sneaking a few, I was fine.*

Clinician: *I’m sorry that she had to do that, you know?*

Patient: *You’re not supposed to, I know, but-*

Clinician: *No, please do. I’m glad she did. Thank her for that. Thank her for me.*

**Encounter #6: Patient-initiated and clinician responded with resistance/boundary-setting**

Patient: *You know, so with me doing extra stuff, I need extra pills.*

Clinician: *Hmm.*

Patient: *So, when last time it was prescribed for 180 pills for three months, I really blew through those.*

Clinician: *Yeah. So unfortunately, I have to stick with that number.*

Patient: *Why?*

Clinician: *For a monthly dosing.*

Patient: *The government and Obama, huh?*

Clinician: *No, not the government and Obama – just regulations on a controlled substance.*

Patient: *So how soon, like say for instance –*

Clinician: *I can only give you a month at a time, basically.*

Patient: *So that’s 180 pills?*

Clinician: *And that’s not Obama. That’s been there for decades.*

## Discussion

We examined 826 patient–clinician encounters to identify the prevalence of substance use conversations and how they are managed. Our results revealed that less than one-fifth of encounters (121; 14.6%) contained content related to controlled substances. Patients initiated these discussions more often than clinicians but this difference was not statistically significant. We also identified that inadequate pain management, medication/addiction concerns, dose escalation, psychosocial concerns, and substance misuse/drug-seeking behaviors were substance use-related risk factors within these data (Table 3).

Despite routine prescribing of controlled substances, such as opioids, in cancer care, our results demonstrate insufficient conversations related to these substances during clinician–patient encounters. Of note, our results were consistent with our central hypothesis that fewer than half of encounters would contain content related to substances – and that fewer than half of encounters would contain adequate management of substance use concerns. Our data converge with some of the chronic pain literature in cancer care, suggesting that clinicians often minimize and invalidate pain experiences (O’Regan et al. 2023; Zhu et al. 2025). Further, oncology clinicians appear to inadequately respond to substance use risk factors (e.g., neutral or dismissive responses). These data suggest the need for educational and institutional support for prescribing clinicians, as well as clear referral pathways to psychosocial clinician and addiction specialists. Specifically, these findings reflect implications for clinician training and risk communication.

The present study’s findings converge with existing literature on substance use and controlled substances during cancer care. For example, in our study, clinicians provided either neutral or avoidant responses to patient-initiated content (Figure 2). Referrals to psychosocial clinicians were rarely made in instances in which the patient expressed psychosocial concerns, including anxiety, mood disturbance, substance misuse, and sleep impairment. For example, in this secondary analysis of audio-recorded encounters, a psychosocial oncology clinician was made once and a pain specialist referral was made once, for a total of 2 referrals. This observation may, in part, be attributed to oncologists’ reluctance to obtain patients’ substance use histories – or to avoid assessments due to fear of stigmatizing the patients (Check et al. 2024). This finding reinforces prior data suggesting that fewer than half of hospice and palliative medicine fellows had adequate knowledge of addiction or felt that their training adequately prepared them to manage opioid misuse, despite 77.2% having seen at least 1 patient with an SUD (Childers and Arnold 2012). Notably, the present study focused on oncology clinicians rather than palliative care specialists. This distinction is important, as oncology clinicians typically receive less training in managing SUDs – an educational gap that may help explain some of the patterns observed in our findings. Finally, our finding in which 121 out of 826 (14.6%) audio-recorded encounters contained substance use-related content converges with existing literature in which few clinicians order urine drug screening (6% ordering rate), despite high rates of aberrant results among those screened (e.g., 54% aberrant results) (Arthur et al. 2016).

### Study limitations

This study has several limitations. First, the data were collected approximately 10 years ago. Clinical practices, regulations, and cultural attitudes surrounding controlled substances (particularly opioids) have evolved considerably over the past decade (Bulls et al. 2021). Shifts in national opioid prescribing guidelines, institutional policies, and clinician awareness may have influenced how clinicians discuss and manage substance use-related issues in cancer care today. However, challenges strongly support continued relevance of these findings (Yusufov et al. 2023, 2024). Specifically, despite policy changes, recent research continues to document substantial variation, challenges, and disparities in opioid prescribing for patients with cancer. This includes recent data suggesting that opioid use among patients and end-of-life has declined substantially between 2007 and 2017; and that rising pain-related emergency department visits suggest that end-of-life cancer pain management may be worsening (Enzinger and Wright 2020). Therefore, while temporal context must be considered, patterns observed in this study still offer valuable insights into longstanding tensions in cancer pain management and substance use communication (Passik and Portenoy 1998; Passik et al. 1998; Starr et al. 2010).

Second, these data possess methodological limitations. Audio recordings do not capture non-verbal communication (e.g., facial expressions, body language), which are critical to interpreting clinical interactions. Relatedly, this study relied exclusively on audio-recorded encounters without integrating additional sources such as medical records, clinician notes, prescription data, or patient self-reports. These methods limit our ability to validate or contextualize the observed communication patterns or substance misuse risk factors. For example, we based our medication data solely on medications that were mentioned during the audio-recorded encounters (Table 2). A key limitation of this study is that we did not formally distinguish between conversations about controlled substances that included verbal expressions of concern (addiction concerns, early refill requests, etc.) versus routine or administrative discussions (e.g., dosage clarification). While we focused on encounters in which substance use-related risk factors were present, we did not penalize clinicians for offering emotionally neutral responses in conversations without risk factors during our coding process. Future research could benefit from a more granular classification of substance-related conversations by risk intensity, to further our knowledge of how clinician response patterns may differ depending on clinical context and perceived level of concern. Of note, a subset of patient contributed multiple encounters, introducing potential non-independence of observations; however, this is unlikely to meaningfully affect results given that most patients contributed a single encounter. Specifically, given the limited number of repeated observations per patient, the contribution of within-patient correlation to variance estimates was expected to be small and as such, clustering was not modeled. Finally, we used a retrospective, observational design, as this was a secondary analysis of pre-recorded encounters. Since this study is observational in nature and does not allow for causal inference, we could not follow up to assess actual clinical outcomes related to identified communication behavioral or risk factors.

Despite its limitations, this study has multiple strengths. First, this study has a unique focus on real-world communication as used by clinicians and patients in audio-recorded outpatient encounters, thus providing nuanced insight into clinical practice rather than retrospective surveys or hypothetical scenarios. Second, we focused on granular coding of initiation and response and thus our coding framework distinguished between initiator (patient/clinician) and response styles (e.g., neutral, avoidant), providing a more comprehensive understanding of interaction dynamics in cancer care contexts (see Figures 1 and 2). Third, this topic remains timely and relevant despite the data being approximately 10 years old, as the issue of controlled substance prescribing during cancer care remains highly relevant. Fourth, this study not only captures dialogue about controlled substances but also links it with patient-level risk factors (e.g., dose escalation, addiction concerns), which can inform future screening and intervention strategies.

### Clinical implications

These findings highlight a critical need for oncology clinicians to improve how controlled substance use and related risk factors in patient encounters are addressed. Limited and often neutral responses to patient-initiated concerns suggest gaps in communication that may contribute to missed opportunities for risk assessment. Clinical training programs should therefore emphasize skills in risk communication, empathic engagement, and appropriate referral pathways to psychosocial and addiction specialists. Institutions may also benefit from developing protocols and structural supports to ensure that substance use concerns are managed consistently and effectively across cancer care settings. By enhancing communication and response strategies, oncology clinicians can better balance the challenges of providing adequate pain management while mitigating risks associated with prescribed controlled substances.

## Conclusions

Future research could build on these findings to deepen our understanding of clinician–patient communication around controlled substances in oncology settings. Studies using contemporaneous data are needed to assess whether communication patterns have evolved in light of policy changes (Bulls et al. 2021), increased scrutiny of controlled substance prescribing (especially opioids), and growing awareness of substance use risks in cancer care (Yusufov et al. 2019). Longitudinal research could also explore how communication about controlled substances and risk factors unfolds over time during the cancer treatment trajectory. Future work may also use multimodal data collection methods (e.g., combining audio recordings with patient and clinician surveys, electronic health record review, etc.). Finally, we need intervention studies to test the effectiveness of communication training for oncology clinicians aimed at improving clarity, robust and appropriate responses to addiction concerns, appropriate responses, and referral generation for psychosocial concerns (e.g., sleep impairment, anxiety, etc.), as well as increased empathy in discussions about pain management and potential controlled substance-related risks.

## Figure 1 Long description

X axis label Initiator. X axis categories Patient, Clinician, Neither, Both. Y axis label number of cases. Y axis range 0 to 50. Bar values: Patient 51, Clinician 33, Neither 24, Both 13.

Navigate back to Figure 1.

## Figure 2 Long description

A stacked bar graph with a legend listing Engaged, Resistant, Avoidant slash Deflecting, Concerned slash Emotional and Neutral. The x axis label is Role. The x axis categories are Patient and Clinician. The y axis label is Count. The y axis range is 0 to 60. Patient stacked bar: Engaged 25, Resistant 3, Avoidant slash Deflecting 12, Concerned slash Emotional 0, Neutral 10, total 50. Clinician stacked bar: Engaged 10, Resistant 2, Avoidant slash Deflecting 3, Concerned slash Emotional 0, Neutral 18, total 33.

Navigate back to Figure 2.

## Table 1 Long description

The table summarizes demographics for 92 unique patients, reporting age as a mean with standard deviation and other characteristics as counts with percentages. Average age was 59.5 years with a standard deviation of 11.8. Sex distribution was 52 female (56.5 percent) and 40 male (43.5 percent). Race and ethnicity were predominantly non-Hispanic White at 74 (80.4 percent), followed by Black at 15 (16.3 percent), with small numbers of Asian (2) and American Indian or Alaska Native (1), and no Hispanic patients. Most patients were married at 67 (72.8 percent); divorced or separated was 16 (17.4 percent), widowed 5 (5.4 percent), and never married 4 (4.3 percent). Education levels were broadly distributed, with completed college the largest group at 32 (34.8 percent), then some college 26 (28.3 percent), and both completed high school or GED and graduate school at 17 each (18.5 percent). One encounter had missing demographic information, so totals may not reflect every encounter.

Navigate back to Table 1.

## Table 2 Long description

The table reports how many encounters mentioned each controlled substance, grouped into opioids, benzodiazepines, and Z-drugs or sleep aids. Among opioids, oxycodone is the most frequently mentioned at 67 encounters, followed by OxyContin at 30. Other opioids are mentioned much less often: Percocet 12, morphine 11, Dilaudid 7, hydrocodone and fentanyl 6 each, Vicodin 5, methadone 3, and tramadol, tapentadol, and Demerol 1 each. For benzodiazepines, Ativan is mentioned in 14 encounters, ahead of Xanax 7, Klonopin 4, and Valium 2. In the Z-drugs and sleep aids category, Ambien is mentioned in 14 encounters, matching Ativan’s count. Counts reflect mentions within encounters and do not indicate dose, duration, or whether the medication was prescribed, taken, or only discussed.

Navigate back to Table 2.

## Table 3 Long description

The table summarizes risk factor content categories in clinical conversations, listing how often each category occurred, who initiated it, example quotes, and the type of response. Inadequate pain management is the most frequent category at 33 cases (27.5%), with both patient- and clinician-initiated examples and responses described as engaged or emotional and concerned. Medication or addiction concerns occur 20 times (16.7%) and show mixed response patterns: one engaged exchange with a therapy referral, one avoidant instance with no response, and one neutral instance where refills are provided. Dose escalation appears 15 times (12.5%) and is met with resistant boundary setting, where the clinician declines increasing the amount. Psychosocial concerns occur 13 times (10.8%); the response quote is provided but the response type is not specified. Substance misuse is least frequent at 5 cases (4.1%) and is paired with an avoidant pattern with no response. Some categories include multiple example rows, and not every row includes a response type, so response-pattern comparisons are illustrative rather than complete.

Navigate back to Table 3.
